# Epidemiology, Molecular Epidemiology and Evolution of Bovine Respiratory Syncytial Virus

**DOI:** 10.3390/v4123452

**Published:** 2012-11-30

**Authors:** Rosa Elena Sarmiento-Silva, Yuko Nakamura-Lopez, Gilberto Vaughan

**Affiliations:** 1 Facultad de Medicina Veterinaria y Zootecnia, Universidad Nacional Autónoma de México, Circuito Exterior, Ciudad Universitaria, Delegación Coyoacán, México, D.F. 04510, Mexico; Email: rosass@unam.mx (R.E.S-S.); 2 Consejo Estatal para la Prevencion y Control del Sida-Centro Ambulatorio para la Prevencion y Atencion del Sida e ITS (COESIDA-CAPASITS) Oaxaca, Mexico; Email: yuko@unam.mx (Y. N.); 3 Instituto de Diagnóstico y Referencia Epidemiológicos, Carpio 470, Col. Santo Tomas, Mexico D.F. 11340, Mexico; Email: gilvaughan@yahoo.com (G.V.)

**Keywords:** BRSV, global distribution, genotypes, evolution.

## Abstract

The bovine respiratory syncytial virus (BRSV) is an enveloped, negative sense, single-stranded RNA virus belonging to the pneumovirus genus within the family Paramyxoviridae. BRSV has been recognized as a major cause of respiratory disease in young calves since the early 1970s. The analysis of BRSV infection was originally hampered by its characteristic lability and poor growth *in vitro*. However, the advent of numerous immunological and molecular methods has facilitated the study of BRSV enormously. The knowledge gained from these studies has also provided the opportunity to develop safe, stable, attenuated virus vaccine candidates. Nonetheless, many aspects of the epidemiology, molecular epidemiology and evolution of the virus are still not fully understood. The natural course of infection is rather complex and further complicates diagnosis, treatment and the implementation of preventive measures aimed to control the disease. Therefore, understanding the mechanisms by which BRSV is able to establish infection is needed to prevent viral and disease spread. This review discusses important information regarding the epidemiology and molecular epidemiology of BRSV worldwide, and it highlights the importance of viral evolution in virus transmission.

## 1. Introduction

The bovine respiratory syncytial virus (BRSV) has been recognized as a pathogen in cattle responsible of an acute respiratory disease syndrome in beef and dairy calves since the early 1970s [1,2]. The impact of BRSV infection on the cattle industry results in economic losses due to the morbidity, mortality, treatment and prevention costs that eventually lead to loss of production and reduced carcass value [3]. 

**Figure 1:**
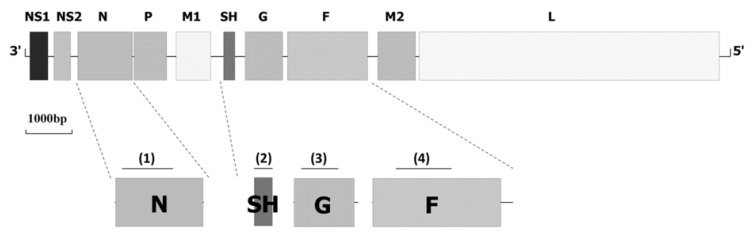
Bovine respiratory syncytial virus (BRSV) genome scheme and commonly used region for molecular epidemiology studies. The areas encoding the BRSV proteins are represented in boxes. Targeting regions are following: region (1), N region (nt 1294 to nt 1984); region (2), SH complete genome (nt 4268 to 4513); region (3), G region (nt 4864 to 5353); region (3), F region (nt 6071 to nt 6812). Nucleotide positions were given in reference to strain NC_001989.

BRSV is an enveloped, non-segmented, negative-stranded RNA virus belonging to the *Pneumovirus* genus within the subfamily *Pneumovirinae*, family *Paramyxoviridae* [4]. The BRSV virion consists of a lipid envelope containing three surface glycoproteins (glycoprotein [G], the fusion protein [F] and the small hydrophobic protein [SH]) (Figure 1). The envelope encloses a helical nucleocapsid composed by the nucleoprotein (N), the phosphoprotein (P), the viral RNA-dependent polymerase protein (L) the M protein and a transcriptional anti-termination factor known as M2-1. The genomic RNA (~15,000 nucleotides in length) also encodes an RNA regulatory protein M2-2 and two non-structural proteins, NS1 and NS2 [4].

BRSV is closely related to human RSV (HRSV), and the epidemiology and pathogenesis of infection between these two viruses share some similarities and also many differences [5]. The similarities between the two viruses have facilitated the unveiling of some of the mechanisms by which BSRV can cause disease. However, the means used by the virus to warrant transmission among individuals within and between herds have remained elusive.

Understanding of the global epidemiology and molecular epidemiology of BRSV has significantly improved over recent years. In this review, we discuss various aspects of the epidemiology and molecular epidemiology of BRSV as well as their relationship with viral evolution.

## 2. Epidemiology of BRSV

BRSV infection is widely spread around the world, most likely as a direct result of the movement of cattle [6]. Regardless of the geographical location, infectivity rates are usually rather high, suggesting that viral transmission is a common event among herds. Cattle are the principal reservoir of infection; however, sheep can also become infected [7]. Intra-herd transmission usually occurs by aerosols, allowing the virus to enter susceptible cattle via the respiratory tract. However, local spread and airborne transmission between herds are not of great importance for inter-herd transmission despite the circulation of BRSV in a given geographical region [8]. On the other hand, direct transmission between herds is frequently a consequence of the introduction of new infected animals, while indirect transmission occurs by individuals visiting farms. Some of the main risk factors for BRSV transmission include large herd size and common farm practices such as not providing boots to visitors and dual-purpose farms [9,10]. Additionally, it has also been proposed that good management and better hygienic routines have a direct impact on overall health status [8].

BRSV outbreaks commonly occur during winter [11]. Thus, clinical disease is commonly diagnosed during autumn and winter in temperate regions [12]. Nevertheless, infection can also be observed during summer [12,13]. The sero-prevalence of BRSV infection varies greatly across different geographical regions [10,14,15,16,17]. The distribution of BRSV is most likely affected by the movement of cattle, as insect vectors are not believed to play a role in viral transmission [6]. The morbidity of the disease is quite high, and in some instances, it has been responsible for up to 60% of the clinical respiratory diseases among dairy herds [13,18]. In general, the frequency of BRSV is strongly associated with cattle population density in the region and with the age of the host [13,19,20]. Interestingly, BRSV infection is also associated with a high morbidity of up to 80% and with mortality that can reach up to 20% in some outbreaks.

BRSV outbreaks can become epidemics affecting animals in all age groups. However, the age distribution of BRSV infection seems to be a function of exposure. In other words, herds that have been previously exposed to the virus tend to experience infections that are limited to younger, more susceptible animals. In consequence, morbidity is commonly high during the occurrence of outbreaks [21]. Importantly, natural infection affects both beef and dairy cattle, although management practices can significantly impact the infectivity rates [22]. Climate also favors the dissemination of the virus during winter, after the sudden drop in temperature [11], although infection can occur throughout the year.

The mechanisms that are responsible for the survival of the virus within a given population are not fully understood. Controversial information has been reported about viral persistence. Nonetheless, chronicity has been proposed as a mechanism that might play role in disease spread [23]. BRSV can be isolated from asymptomatic animals and can persist for several months [6]. Thus, one possibility is the existence of persistently infected calves, which might start shedding the virus under specific conditions [24,25]. Therefore, latent infection among herds might occur, providing a possible explanation for the occurrence of outbreaks among relatively isolated calves. However, some reports have suggested that subclinical infection in cattle is not a plausible mechanism for the persistence of BRSV in dairy herds [26].On the other hand, clinically ill animals are believed to be the most likely sources of infection, and therefore, the most likely explanation for recurrent infections is the reintroduction of the virus into the herd before the occurrence of a new outbreak. This controversial issue requires more detailed studies aiming to assess the role of persistence in virus spread to fully understand the mechanism exploited by the BRSV to warrant transmission.

The prevalence of the disease varies greatly in North America. For instance, in the United States, original studies conducted during the early 1970s showed a frequency of infection of 67% among adult cattle [27]. However, in a few instances, 100% of the animals within herds showed the presence of specific antibodies. Subsequently published reports showed that up to 81% of herds had specific antibodies against BRSV [28,29]. Additionally, the incidence of sero-conversion soon after the occurrence of an outbreak has been reported to be as high as 45% [30]. Furthermore, the sero-positivity rate among asymptomatic cattle has been shown to reach up to 95% [15]. Importantly, the sero-positivity rate seems to be closely associated with the age of the host, showing a higher prevalence among older animals. Moreover, southern regions of the U.S. commonly exhibit a higher sero-prevalence than the northern parts of the country [31]. These discrepancies could be explained by differences in the vaccination practices in those regions, as well as by management practices or by sampling errors. Thus, the frequency of BRSV infection in a particular region is subject to a number of factors that can drastically change the prevalence of the disease. Interestingly, the mortality among herds experiencing respiratory disease in the U.S. can reach up to 13% [32]. While other viruses, such as the bovine viral diarrhea and parainfluenza viruses, could also account for the elevated mortality rates seen in the U.S., BRSV remains as a very important ethological agent and a probable cause of death due to respiratory disease in cattle.

In Canada, initial reports have shown that the overall frequency of BRSV infection might be nearly 36% [16,17]. However, during outbreaks, the percentage of sero-positive individuals within a herd can range between 22% and 53%. Subsequent studies confirmed the high frequency of the disease (40%) among feedlot calves [33]. Similar to what has been observed in the U.S., BRSV is still an important cause of mortality among cattle in Canada [34]. In Mexico, the circulation of BRSV was recently reported in two different regions of the country [35,36]. Both studies reported rather high overall frequencies of BRSV infection (52% and 90.8%). The reasons for the significant differences in the distribution of BRSV infection between the two regions are not known; however, age played a major role in the distribution of infection. Additionally, BRSV has also been shown to circulate in other regions of the Americas [10,37,38].

In Europe, shortly after the virus’s discovery, it was reported to have widely circulated in different parts of the continent [39]. In Sweden, different studies have shown high frequencies of antibodies in milk, ranging between 41% and 89% depending on the geographical region of the country [13]. In general, higher antibody frequencies in milk samples were observed in samples from the southern regions of Sweden, while lower frequencies were detected in samples from the northern part of the country. The authors attributed the discrepancies observed among differences herds to cattle population density, as highly populated regions were more common in the southern part of Sweden. More recently, the geographical distribution of BRSV in the country has been studied [40]. The aforementioned study showed that infection with BRSV occurs predominantly in the central-western and southern parts of Sweden. In particular, two regions of the country, Skaraborg and Skåne, displayed a high prevalence of BRSV infection. Similarly, Danish reports have also shown rather high frequencies of BRSV infection (54%) [41]. Likewise, a high prevalence of BRSV infection has been observed in Belgium [42]. Circulation of the virus in Scotland has also been reported, although conclusive figures regarding BRSV prevalence are not available [43]. Furthermore, the presence of antibodies in bulk tank milk samples has been reported in herds from England [44]. Additionally, a high sero-prevalence of BRSV has also been reported in Northern Europe [21,45].

In Africa, Ethiopia and South Africa have also been shown to have high incidences of BRSV infection [14,46,47,48]. Other countries, in different regions, such as Turkey, have also been shown to a have high sero-prevalence, which can reach up to 43% [49]. Unsurprisingly, high sero-prevalence has also been associated with large-capacity facilities, rather than with small farms. Interestingly, organic farms have been shown to exhibit lower antibody prevalence when compared to conventional farms [22]. These findings highlight the importance of management for the effective control of viral transmission and disease spread, which are closely associated with different farming methods

Thus, more information is required to understand the mechanisms that allow for viral survival in a given geographical region. Monitoring of outbreaks among herds is likely to provide valuable information that might help us to understand in greater detail the epidemiology of BRSV infection.

## 3. Molecular Epidemiology and Evolution of BRSV

Limited information is available regarding the molecular epidemiology of BRSV infection. However, the rapidly changing field of DNA sequencing might help to unveil the molecular mechanisms exploited by BRSV to assure transmission. Additionally, the field of phylogenetics has contributed to identify the existence of diverse BRSV genotypes and their genetic relationship while helping understand the molecular basis of BRSV genetic history [50,51]. Phylogeny approaches have also been particularly useful for studying the origins and subsequent evolution of BRSV [51]. Recent advances in phylogenetics have allowed for the analysis in greater detail of sequence information, which could also help to understand the patterns of BRSV infection [52]. Additionally, current phylogenetic methods facilitate the estimation of the time to the origin of a new viral strain, of the emergence of new species, identification of viral recombination, of population size, and of how the virus spread and evolve in particular settings [53]. Thus, the information obtained from phylogenetic studies could assist in the design and implementation of measures aimed to prevent BRSV transmission. Molecular epidemiological approaches have provided important insights into BRSV evolution, spread and transmission [50,54]. Phylogentic analysis have also helped to identify the presence of mutations mapping along the immunodominat region located on the G protein [55] and to elucidate the origin circulating strains in certain geographical regions [56]. Thus, the study of the molecular epidemiology of the virus will most likely improve our understanding of the dynamics of virus transmission and help us to implement informed public health policies.

The occurrence of antigenic variation among BRSV isolates was first suggested by the lack of reaction between a polyclonal serum made against one particular viral strain, which failed to recognize a different isolate [57]. Furthermore, viral heterogeneity was also inferred from the differences observed among the molecular sizes of some of the structural proteins, which implied that BRSV was composed of distinct subgroups [58,59]. The existence of diversity among BRSV isolates was further assessed by the reactivity of a set of strains against anti-HRSV monoclonal antibodies [58]. In that pioneering study, the authors showed the existence of different recognition patterns among different BRSV isolates, thus implying antigenic diversity. The aforementioned findings highlighted the limitations and efficacy of the BRSV vaccine, suggesting that vaccine failure could be at least partially attributed to a possible broader antigenic spectrum of the BRSV population [58]. Thus, these initial reports suggested the presence of two distinct BRSV subgroups. These observations were later confirmed by another study [60]. Estimation of BRSV antigenic variation further confirmed the presence of two major and one intermediate subgroups [61]. Currently, four antigenic subgroups (A, B, AB, untyped) have been identified in BRSV; however, they might only represent variants of a single major antigenic group [62].

The study of the molecular epidemiology of BRSV began with the identification of the nucleotide sequences of the glycoprotein (G), fusion (F), nucleocapsid (N), matrix (M), phosphoprotein (P), small hydrophobic (SH) and M2 proteins during the early 1990s [55,63,64,65,66,67]. The initial assessment of the homology along the P protein between two isolates showed an identity of 97% at the nucleotide level [67]. Subsequent studies, which analyzed the nucleotide variation of a limited number of isolates at the G protein level, showed that the levels of identity between BRSV strains ranged between 84% and 95% [60,68]. This degree of heterogeneity was suggestive of only one single genetic group. The first direct comparison between the antigenic heterogeneity and molecular diversity among BRSV isolates showed that the antigenic divergence observed among BRSV strains was the result of inter-subgroup variation [60]. The characterization of the antigenic structure of the BRSV G protein provided further information about the mutations responsible for the distinctive BRSV groups reported at that time [69]. Subsequent studies further confirmed the existence of antigenic divergence and antigenic variability among wild BRSV isolates [70]. The partial nucleotide sequences of the G protein from a set of isolates, along with the recognition pattern by monoclonal antibodies against the BRSV G, F, N, and P proteins, showed random antigenic differences among the isolates, although cross-reactivity to the viral protein epitopes was observed, particularly with the F protein. Moreover, structural differences between the F and P proteins were also observed. The P protein exhibited diverse patterns, due to differences in molecular size, when subjected to polyacrylamide gel electrophoresis analysis. However, the structural differences along the P protein were not correlated with the antigenic differences observed in the F and N proteins. Overall, the nucleotide sequence identity in the G protein ranged from 94.1% to 99.9%. In comparison, the predicted amino acid sequence homology ranged from 89.9% to 99.6%, supporting the theory that BRSV belonged to a monophyletic group [70].

More comprehensive studies, which analyzed the reaction patterns against anti-BRSV G protein and the genetic diversity occurring in a larger segment of the G protein gene from several isolates obtained from different geographical regions, showed that the intragroup genetic variation among BRSV strains ranged between 88% and 100% [71]. The corresponding phylogenetic analysis revealed the presence of two main branches. Branch I was further subdivided into two groups, Ia and Ib, and then into five different lineages, each representing a geographic cluster. Thus, group Ia contained strains belonging to the antigenic subgroup A, whereas branch Ib consisted of strains of European origin belonging to the subgroup AB. Branch II, in contrast, grouped all viral strains classified as antigenic subgroup B. A third independent cluster included a set of Scandinavian strains that were not grouped with any of the aforementioned branches. A direct correlation was also observed between the positions along the phylogenetic tree and some of the strains and their antigenic patterns. Thus, this study can be considered to be the first research demonstrating that BRSV belongs to a single antigenic group with different genetic variants [71]. A subsequent study demonstrated that isolates of Danish origin formed three distinctive lineages within a separate cluster. These isolates were also closely related to the 220-69Bel strain, the prototype strain of the intermediate antigenic group [72]. Interestingly, viral isolates from the Czech Republic were closely related to the Danish strains isolated during the mid-1990s, a finding that was most likely due to animals being imported into the country, rather than due to virus evolution [73]. Moreover, a recently comparative analysis between Swedish and Danish isolates showed that the reduced sequence diversity among Swedish strains might be due to the relatively closed cattle population in Sweden as a consequence of the limited import of cows into the country [12]. In conjunction, these findings support the participation of import of cows in the increase of nucleotide sequence diversity of BRSV lineages in given regions.

**Figure 2:**
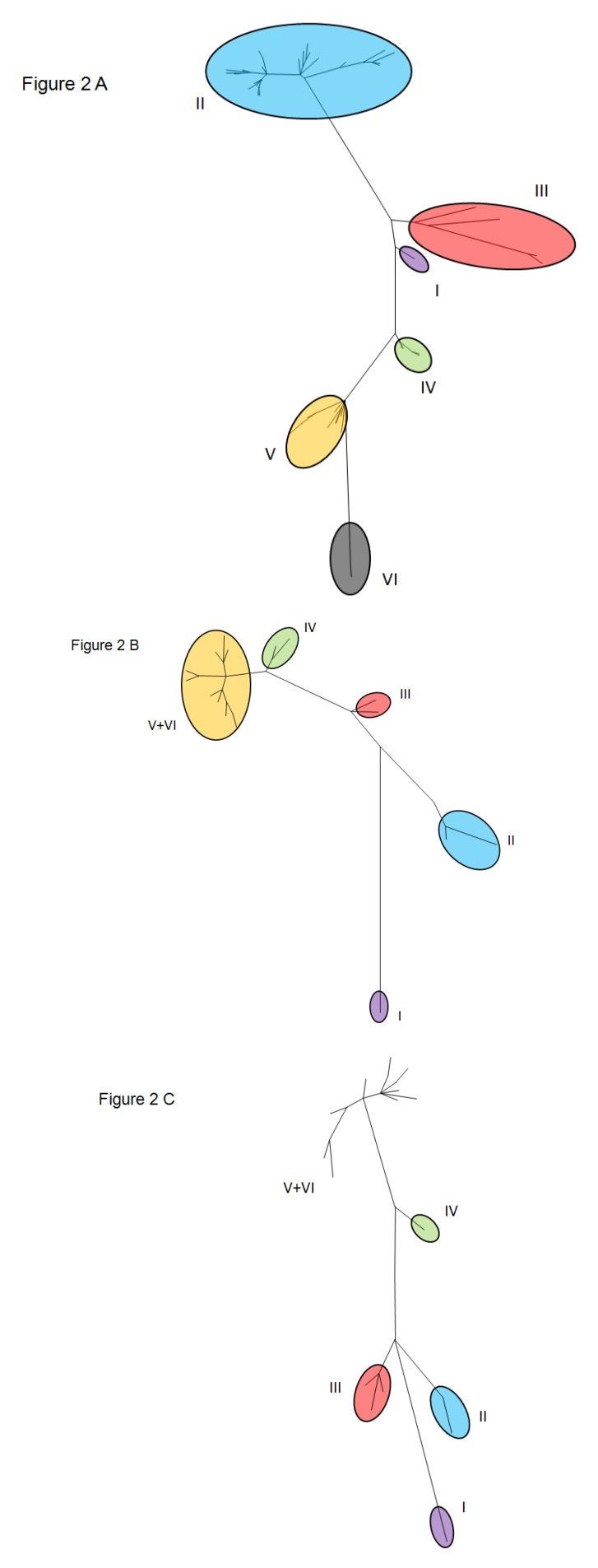
Phylogenetic analysis of BRSV genomic regions. Maximum likelihood phylogenetic trees from the G (A), F (B) and N (C) proteins were generated using representative strains from each BRSV genotype [50].

A large-scale study addressing the global molecular epidemiology and evolution of BRSV included 54 European and North American isolates, in addition to previously reported sequences [50]. The study assessed the diversity of the N, F and G protein nucleotides and analyzed amino acid sequences, as well as their phylogenetic relationships. The average percentage of pairwise divergences was lowest for the N and F protein genes (2%), in comparison to the G protein gene (8%). In general, complete homology was observed among all of the BRSV proteins among animals within the same herd. A limited number of differences were detected within the N and G protein gene sequences among a few herds. This finding suggests that a single virus or a group of very closely related viruses would seem to infect predominantly a given herd at a given time [74]. Phylogenetic analysis, based on the G protein sequence, classified the isolates into six different groups (Figure 2A) [50]. The topology of the phylogeny was retained when the analysis of the N and F protein gene sequences was conducted; however, only five phylogenetic groups were observed for either protein (Figure 2B and 2C) [50]. As expected, the characteristic clustering of BRSV sequences, according to geographical origin and date of isolation, was observed, further supporting the theory of geographical and temporal clustering. Subgroup I consisted of old European strains isolated before 1976. Subgroup III included viruses exclusively from the United States. Subgroups II, IV, V, and VI were composed entirely of “younger” European isolates. Strains from northern Europe, Denmark, and Sweden were clustered in subgroup II, while those from the Netherlands, Belgium, and France were found in subgroups II, IV, V, and VI [75]. Interestingly, the study identified vaccine failure among animals harboring infections with BRSV groups V and VI, indicating that commercial vaccines performed poorly against infections by such viral groups. Moreover, continuous evolution of the BRSV N, G, and F proteins was also observed, which seemed to be correlated with the implementation of vaccination in different countries. Moreover, strong, positive selection was shown on the mucin-like region of the G protein and on particular sites of the N and F proteins. The analysis of French BRSV isolates included in this study also showed the presence of mutations located along the conserved central hydrophobic part of the ectodomain of the G protein, resulting in the loss of four Cys residues, two disulfide bridges and, consequently, a helix, which is critical for the three-dimensional structure of the G protein. These observations suggested the continuous modification of the highly conserved central region of the immunodominant G protein, thus highlighting the importance of considering BRSV evolution in the rational development of vaccines [50]. Overall, this work could be considered as the foundation of modern BRSV molecular epidemiology, it has also established the basis for subsequent studies looking at the molecular epidemiological patterns of BRSV infection in different geographical regions [37,56,76,77].

Importantly, BRSV intrahost population has been shown to exist as a complex mixture of viral variants, ambiguously referred as “quasispecies” [51]. Analyses of the BRSV G protein gene have demonstrated the spectrum of subpopulations co-existing in clinical isolates. Clonal analysis revealed the nucleotide heterogeneity along the G-coding region, exhibiting mutation frequencies ranging from 6.8x10^4^ to 10.1x10^4^ substitutions per nucleotide. These findings suggest that BRSV populations evolve as complex and dynamic mutant swarms, despite the virus’s apparent genetic stability.

Molecular epidemiology approaches have shown the circulation of identical viruses among animals within the same herds, especially during the occurrence of outbreaks [74]. However, viral strains from recurrent outbreaks have varied significantly (up to 11%), suggesting the circulation of different BRSV viral variants that can persistently infect calves within a herd. In consequence, and because of intrahost viral evolution, new, highly fit viruses became dominant and spread from a single, or a few, animals associated with each new outbreak. Alternatively, and based on the high level of diversity observed between outbreaks, this finding might suggest that BRSV outbreaks are the result of the introduction of new viral strains into the population [74]. Interestingly, the reduced exposure to new BRSV strains has been shown to limit the diversity of the circulating BRSV population [12]. Importantly, all aforesaid studies have focus on the nucleotide sequence of the gene coding for the G protein. While the information obtained from the G protein has been extremely valuable to understand the landscape and sequence space available to BRSV, the somehow limited sequence information provided by this region significantly handicaps our capacity to fully characterize viral strains. Future studies analyzing longer sections of the BRSV, and possibly the full-length of the viral genome, will likely provide a more accurate picture of the viral strains circulating worldwide.

The global molecular epidemiology of BRSV has remained elusive. Only sparse reports addressing the distribution of BRSV groups are available in the literature. Consequently, the molecular characterization of BRSV isolates from representative regions of the world is still rather incomplete. A study conducted in Japan in the early 2000s reported the circulation of strains belonging to subgroup III. The isolates were further subdivided into two distinctive lineages [76]. Unfortunately, this study only accounted for a very limited number of isolates, which notably precluded us from fully understanding the molecular epidemiology of BRSV in the region. More recently, two Brazilian studies identified a handful of isolates belonging to BRSV group B [37,77]. Likewise, this information was limited, and the inference of the molecular epidemiology of BRSV in the country was not possible. Recently, a Swedish report was published describing the molecular epidemiology of BRSV from recent outbreaks (2007**–**2011) occurring among 30 different herds [12]. The report highlighted the circulation of BRSV strains belonging to subgroup II, for the most part, in the southern region of Sweden. A second study showed that isolates from England are related genetically to U.S. strains. [78]. Thus, both reports suggest that live cattle importation plays an important role in the global molecular epidemiology of BRSV.

In summary, the study of the molecular epidemiology of BRSV has evolved considerably over the past two decades. The information generated by the study of the molecular characterization, phylogenetics and evolution of BRSV strains have broadly advanced our understanding of the molecular mechanisms controlling virus transmission and disease spread. However, this field requires extensive research to unveil the means exploited by the virus to attain persistence in a given population. The establishment of molecular surveillance of BRSV in different geographical regions will likely improve the identification of outbreaks, resulting in the implementation of preventive measures aimed to control the disease. The advent of next-generation sequencing platforms on the eve of the “DNA-sequencing era” could also provide a unique opportunity for the discovery of the underlying processes responsible for viral replication and survival in the host.

## 4. Concluding Remarks

BRSV has been recognized as an important cause of respiratory disease in cattle for nearly four decades. The characteristic heterogeneity of the viral genome and its low fidelity in replication are some of the most important features that the virus exploits to assure its survival and persistence within the host. The instability of the viral particle usually leads to unsuccessful attempts to isolate viral strains in the laboratory from clinical specimens. Consequently, molecular approaches are rapidly becoming the gold standard for the correct identification and characterization of BRSV in clinical cases. As result, the field of the molecular epidemiology of BRSV has gained significant strength and has further enhanced our knowledge about BRSV distribution and transmission patterns worldwide. The arrival of new and more sophisticated molecular methods, including next-generation sequencing, will most likely help to unveil the genetic makeup of the circulating viral population in different geographical regions, as well as the mechanisms on which the virus relies for survival, persistence and transmission.
